# Modified stepwise method with the guidance of QDOT MICRO catheter for mitral isthmus ablation in patients with persistent atrial fibrillation

**DOI:** 10.3389/fcvm.2026.1780048

**Published:** 2026-02-26

**Authors:** Jingchao Li, Chaoyu Zhao, Luqian Cui, Xin Wang, Qianqian Feng, Huihui Song, Cong Ding, Hailan Wang, Haijia Yu, Yingjie Chu, Shujuan Dong

**Affiliations:** 1 Department of Cardiology, Henan Provincial People's Hospital, Zhengzhou University, Zhengzhou, China; 2 Henan University People's Hospital, Henan provincial People's Hospital, Zhengzhou University, Zhengzhou, China; 3 Department of Cardiology, Henan Provincial Chest Hospital, Zhengzhou University, Zhengzhou, China; 4 Department of Cardiology, Zhoukou Central Hospital, Zhoukou, China

**Keywords:** catheter ablation, mitral isthmus, persistent atrial fibrillation, QDOT MICRO catheter, vein of Marshall

## Abstract

**Background:**

Ethanol infusion of the vein of Marshall (EI-VOM) has elevated the success rate of mitral isthmus (MI) block in patients with persistent atrial fibrillation (PeAF). However, the procedure involve the extensive endocardial ablation and epicardial ablation, which brought the operational difficulty and risk.

**Material and methods:**

Patients with PeAF were randomly assigned in a 1:1 ratio to either STSF catheter group using the stepwise method (STSF group) or QDM catheter group using a modified stepwise method (QDM group) for MI ablation. The modified stepwise method was as follows: step 1, The potential of VOM was mapped from endocardium using the QDM catheter. Step 2, EI-VOM. Step 3, precise endocardial ablation guiding by VOM potential. Step 4, QDM catheter was cannulated into the CS for epicardial ablation, especially the ostium of Marshall. The immediate procedural results were compared between the two groups.

**Results:**

After excluding 5 patients with unsuccessful EI-VOM, 68 patients were divided into STSF group (36 cases) and QDM group (32 cases). The potential of VOM could be clearly mapped from endocardium using a QDM catheter. Both the accumulated operation time (*p* = 0.032) and ablation time (*p* < 0.001) were significantly shorter in the QDM group compared to the STSF group. QDM group achieved more conduction blocks of MI after a single endocardial line ablation (71.9% vs. 36.1%, *p* = 0.017) with fewer ablation points (*p* < 0.001) compared to the STSF group. The block rate of the MI after endocardial ablation alone was also higher in the QDM group than in the STSF group (90.6% vs. 69.4%; *P* = 0.019), which avoided epicardial ablation. Even if epicardial ablation is necessary, the number of ablation points on the epicardial surface in the QDM group would be fewer than in the STSF group (*p* < 0.001).

**Conclusions:**

The QDM catheter can be used to map the potential of VOM from endocardium, thereby facilitating precise endocardial mitral isthmus linear ablation. The modified stepwise approach effectively reduces the number of endocardial ablation points and the likelihood of requiring epicardial ablation. (NCT06145906, ClinicalTrial.gov).

## Introduction

Pulmonary vein isolation (PVI) has become the cornerstone of atrial fibrillation (AF) ablation, but it is not enough to maintain sinus rhythm for persistent atrial fibrillation (PeAF) (1, 2). Additional linear ablation beyond PVI, originating from the Cox-Maze surgical technique, is recommended to enhance the success of the ablation. Recently, authoritative journals have reported that the PVI plus linear ablation strategy is superior to PVI alone for patients with PeAF, especially for those facilitated by ethanol infusion of the vein of Marshall (EI-VOM) (3–5). Mitral isthmus (MI) ablation is a key component and the most difficult step to achieve bidirectional block in the linear strategy due to its anatomical structure (6). The success rates of MI block have been reported to be variable in different studies, and the high block rate always involved extensive endocardial ablation and epicardial ablation (7–9). The imprecise ablation strategy increased the operational difficulty and risk. Our previous study introduced an efficient stepwise strategy for MI block with a high block rate; however, the procedure also requires two lines of endocardial ablation and epicardial ablation in more than 20% of cases (7).

A previous study reported that the potential of the vein of Marshall (VOM) could be recorded by a special mapping catheter (10). The novel QDOT MICRO (QDM) catheter, with three microelectrodes inserted at the top of the catheter, was used to provide high-resolution intracardiac mapping and enhance the ability to precisely detect conduction gaps (11). Therefore, the potentials originating from the Marshall ligament may be recorded from the endocardium using the QDM catheter, which can facilitate precise endocardial ablation.

This study aimed to take advantage of the QDM catheter, recording potentials from VOM on the endocardial surface, to formulate the endocardial ablation line precisely, thereby increasing the success rate of MI block from the endocardium and reducing the possibility of ablation from the epicardium.

## Methods

### Study population and study design

This study was a prospective, single-center, open-label, randomized study conducted at Henan Provincial People's hospital. We enrolled 73 consecutive patients (aged 18–75 years) with PeAF for more than 1 year who underwent first-time catheter ablation in our hospital between October 3, 2024, and February 28, 2025. The patients were randomly categorized into the STSF ablation catheter (Biosense Webster, Irvine, USA) group (*n* = 37) using the stepwise method we proposed before and into the QDM catheter group (*n* = 36) using the modified stepwise method for MI ablation (Supplementary Figure S1) (8). The numerical table method was used for randomization. Patients with previous cardiac surgery, left atrial diameter more than 55 mm, or left ventricular ejection fraction (LVEF) less than 35% were excluded. The trial was approved by the Institution Review Board of our hospital (permission no: 2021092).

### Preoperative preparation and intra-procedural setting

All the included patients underwent cardiac contrast-enhanced computed tomography or transesophageal echocardiography to rule out left atrial thrombosis. Antiarrhythmic drugs (amiodarone or others) were discontinued for a minimum of five half-lives before the ablation procedure.

All the procedures were performed under general anesthesia. A steerable decapolar catheter (DecaNAV; Biosense Webster, Irvine, USA) was used to construct the matrix and advanced into the CS. Then, the CS was mapped using the DecaNAV catheter. The first transseptal puncture was performed under the guidance of x-ray and intracardiac echocardiography. A Pentaray catheter (Biosense Webster, Irvine, USA) was inserted into the left atrium (LA) using the Swartz sheath (Abbott, Chicago, USA), and electro-anatomical maps of the pulmonary veins (PVs) and LA were constructed. The ablation strategy of pulmonary vein isolation (PVI) plus linear ablation based on EI-VOM was chosen. Patients with absent or abnormally coursing VOM (near the left atrial appendage or posterior wall, separated from the mitral isthmus) were excluded. The ablation sequence was as follows: EI-VOM, PVI, LA roofline, MI, bottom line, and cavotricuspid isthmus. The posterior wall of PVI and roof line ablation were performed using the very high-power short-duration (vHPSD) model (90W) of the QDM catheter in the QDM group. The other positions’ ablation in the QDM group and the whole ablation procedure in the STSF group were performed using the conventional-power temperature-controlled (CPTC) model (45W). The ablation procedure for MI followed the two workflows below according to different groups.

### Workflow of the stepwise ablation method

The detailed steps of the stepwise method for MI block using the STSF ablation catheter were according to the article we published previously (8). Briefly, the four steps include EI-VOM, “V-shape” endocardial linear ablation, earliest activation sites (EASs) near the ablation line on the endocardium, and key ablation targets (KAT) in coronary sinus (CS).

### Workflow of the modified stepwise ablation method

The steps of the modified stepwise strategy for blocking MI according to the potentials from Marshall on the endocardial surface recorded by the QDM catheter were as follows:

Step 1. Mapping the potentials of VOM

**Figure 1:**
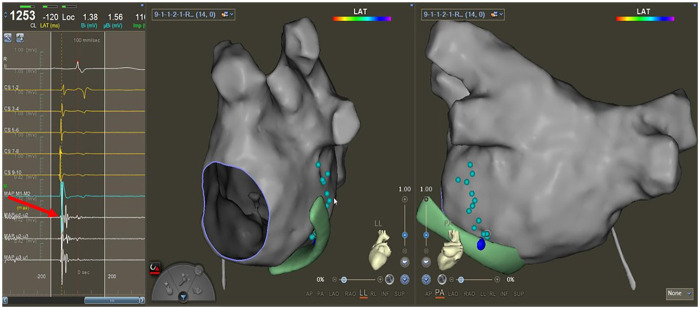
Mapping the potentials of VOM. The trend of these cyan dots could reflect the curve of VOM from the endocardial side. The red arrow points to the special potential (double potentials). The green model is the CS construction. The big blue dot is the ostium of VOM, marked from the epicardium.

After constructing the PVs and LA, sinus rhythm (SR) was restored by transthoracic cardioversion. A 6F Judkins R4.0 guiding catheter was cannulated inside the Swartz sheath to perform CS venography and identify the ostium of VOM. Then, the QDM catheter was sent to the ostium of VOM to record the local potential and mark the position of the VOM ostium on the 3D model of CS precisely. After that, the QDM catheter was sent to the left atrium to detect VOM potentials around the MI area from the endocardial surface, referencing the course of VOM which was guided by CS venography. The site with double potential was marked by special color dots on the 3D model of LA (Figure 1). The course formed by these special dots would be used to guide operators in formulating the first ablation line on endocardial surface.

Step 2: EI-VOM

**Figure 2:**
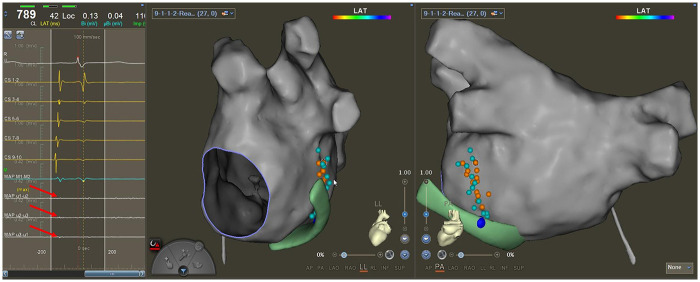
Second mapping of VOM potentials. Little cyan dots represent the locations where potentials could be recorded from VOM before EV-VOM, little orange dots are areas where the QDM catheter was relocated after EV-VOM, and the local potentials (red arrows) were recorded by the microelectrodes on the tip of the QDM catheter. The green model is the CS construction. The big blue dot is the ostium of VOM, marked from the epicardium.

EI-VOM was performed according to a previously published protocol (8). An over-the-wire angioplasty balloon (Emerge 1.5–2.5 mm × 6–8 mm; Boston Scientific) preloaded with a guidewire was advanced into the proximal VOM, and 5–8 mL of 98% ethanol was injected into the VOM for 1 min. After conducting EI-VOM, the QDM catheter was relocated again to the sites marked in step 1 to detect the local potential (Figure 2), and voltage mapping of LA for the second time was performed to locate the low voltage region of MI and the ethanol-induced scar (Supplementary Figure S2).

If the endpoint of bidirectional block was not achieved after EI-VOM, we proceeded to the next step.

Step 3: Endocardial ablation according to the guidance of bipotential recorded by QDM catheter

**Figure 3:**
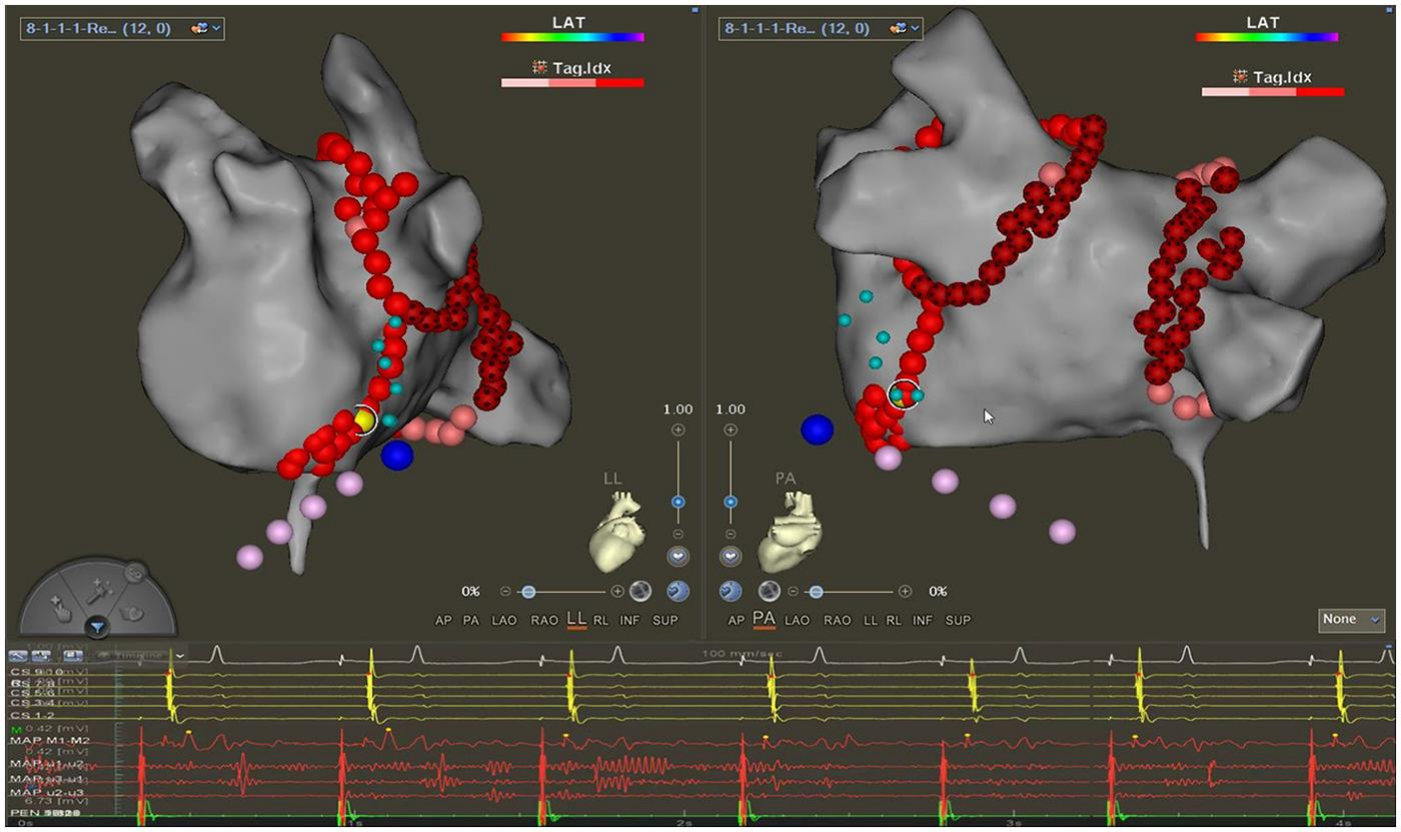
Key ablation targets. The yellow dot is the KAT in the endocardium on the lower line of the “V-shape.” The little cyan dots are locations where potentials could be recorded from VOM in step 1 by the QDM catheter. Pink dots are electrode marks of DecaNAV. The big blue dot is the ostium of VOM, marked from the epicardium. The right: left lateral position. the left: posterior anterior position.

The course of the first ablation line was infinitely close to the line formulated in step 1 according to the double potentials. The ablation points that change the sequence of CS temporarily or permanently and/or prolong the conduction time during ablation were marked and designated as key ablation targets (KATs) (Figure 3) (8). The reinforced ablation of KATs was performed. If MI block wasn't fulfilled, the sequences of potentials on the QDM and CS at the same level were compared to verify whether the MI had been blocked from the endocardium. If the potential on the endocardium was earlier, the second ablation line of “V-sharp” and the ablation of earliest activation sites (EASs) around the two “V-shape” ablation lines were performed. If the block still hadn't been achieved, step 4 would be processed.

Step 4: Precise epicardial ablation

**Figure 4:**
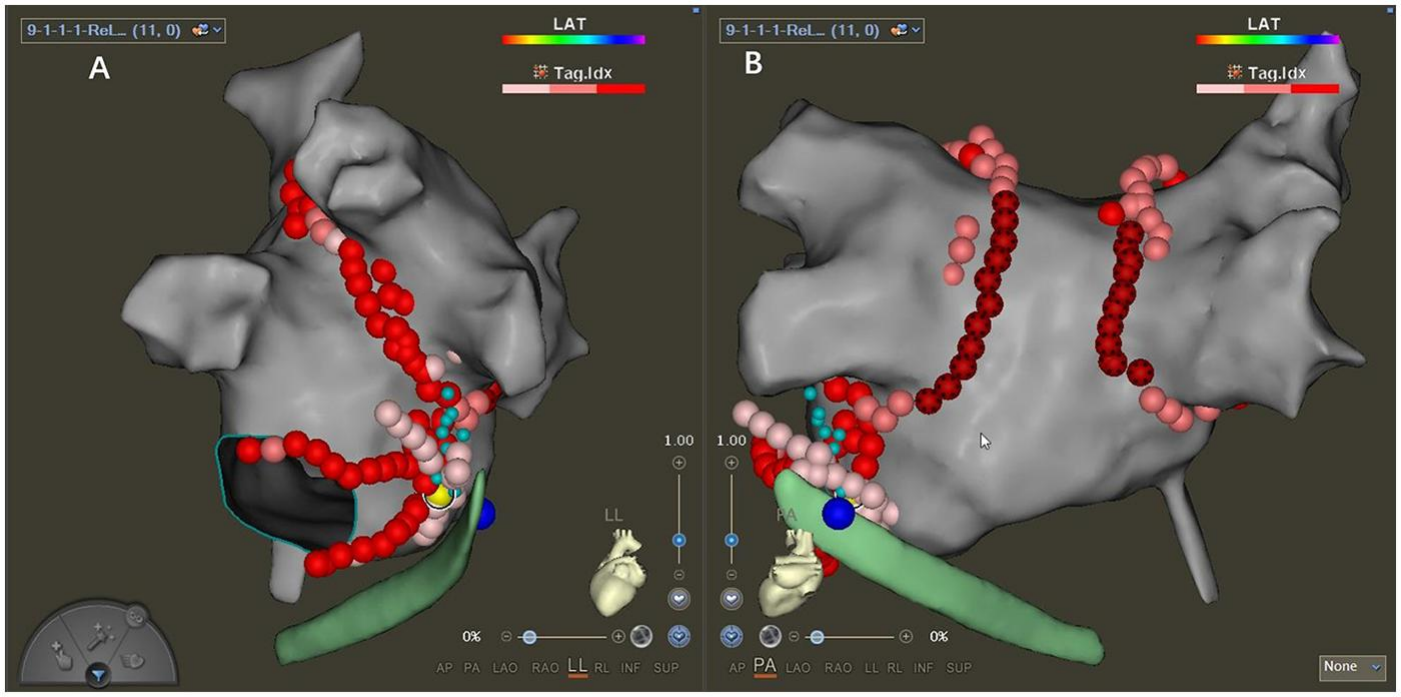
Precise epicardial ablation. The yellow dot is the KAT in the epicardium. The little cyan dots are locations where potentials could be recorded from VOM in step 1 by the QDM catheter. The green model is CS mapped by DecaNAV. The big blue dot is the ostium of VOM, marked from the epicardium. **A**: left lateral position. **B**: posterior anterior position.

The QDM catheter was inserted into the CS to perform precise epicardial ablation. Firstly, ablation around the VOM ostium was performed. Secondly, the anatomically corresponding sites of KATs on the epicardial side were ablated. If the block was not achieved, the ablation scale would be expanded appropriately in the CS (Figure 4).

If the MI block was achieved in step 4, it would be verified again after 20 min to confirm the continued existence of the bidirectional MI block. If the endpoint was not reached, the procedure was terminated and the MI block was considered a failure.

### Definition of MI bidirectional block

Conduction block of MI was assessed by the activation sequence along the CS catheter and the ablation catheter. The conduction block was characterized as an activation detour when pacing on one side of the ablation line. The endpoint of the MI ablation was the achievement of a bidirectional block.

If the Pentaray catheter was used for LAA pacing, proximal-to-distal activation on the CS catheter was considered a unidirectional block. When the QDM catheter was located on the high lateral side of the ablation lines for recording potential sequences and the CS was used for pacing, distal-to-proximal activation of QDM represented a bidirectional block of MI.

Complications related to this procedure were recorded. Furthermore, we calculated the block rate and operation time for each step.

### Statistical analysis

Statistical analyses were performed using the SPSS 25.0 software (SPSS Inc., Chicago, USA). For continuous variables, normally distributed data were expressed as the mean ± standard deviation (SD) and non-normally distributed data were expressed as median [interquartile range (IQR)]. The dichotomous variables were expressed as *n* (%). The normality of data distribution was tested using the Shapiro–Wilk test. Parametric tests (Student's t-test) or non-parametric tests (Mann–Whitney U test) were used for comparing the continuous variables. Categorical variables were compared using the X^2^ test or Fisher's exact test. Two-sided *P*-values were reported and the significance level was set at ≤0.05.

## Results

### Baseline characteristics of patients

The basic characteristics between the two groups did not show any significant statistical differences (Table 1).

**Table 1:** Demographic characteristics of the PeAF patients.

Characteristics	STSF group *N* = 36	QDM group *N* = 32	*P*-value
Age in y, mean (SD)	62.1 ± 23.2	56.9 ± 13.2	0.448
Male, *n* (%)	23 (63.9)	18 (56.3)	0.413
BMI (kg/m^2^)	25.8 ± 4.6	25.9 ± 2.6	0.357
Duration of AF (months)	28.6 ± 14.9	24.2 ± 15.8	0.544
Risk factors, *n* (%)			
Hypertension	21 (58.3)	20 (62.5)	0.726
Diabetes	14 (38.9)	14 (43.8)	0.684
Strock-TIA	7 (19.4)	5 (15.6)	0.680
Coronary heart disease	4 (11.1)	6 (18.8)	0.375
Congestive heart failure	9 (25.0)	3 (9.4)	0.092
Antiarrhythmic drugs, n (%)			
Amiodarone	15 (41.7)	15 (46.9)	0.666
*Β*-block	15 (41.7)	16 (50.0)	0.491
NYHA functional Class≥2, *n* (%)	4 (11.1)	4 (11.8)	0.859
CHA2DS2-VASC Scores	2.08 ± 1.3	2.03 ± 1.3	0.472
Left atrial diameter (mm)	46.5 ± 5.3	45.3 ± 5.9	0.568
Left atrial volume (mL)	156.3 ± 28.0	161.6 ± 28.5	0.996
Left ventricular ejection fraction (%)	52.7 ± 3.5	52.0 ± 4.7	0.102

AF, atrial fibrillation; BMI, Body Mass Index; CHF, congestive heart failure; TIA, transient ischemic attack; CHA2DS2-VASc: Congestive heart failure, Hypertension, Age ≥ 75 years (doubled), Diabetes mellitus, Stroke/transient ischemic attack/thromboembolism (doubled), Vascular disease (prior myocardial infarction, peripheral artery disease, or aortic plaque), Age 65 to 74 years, Sex Category (female).

### The characteristics of potential from marshall

**Figure 5:**
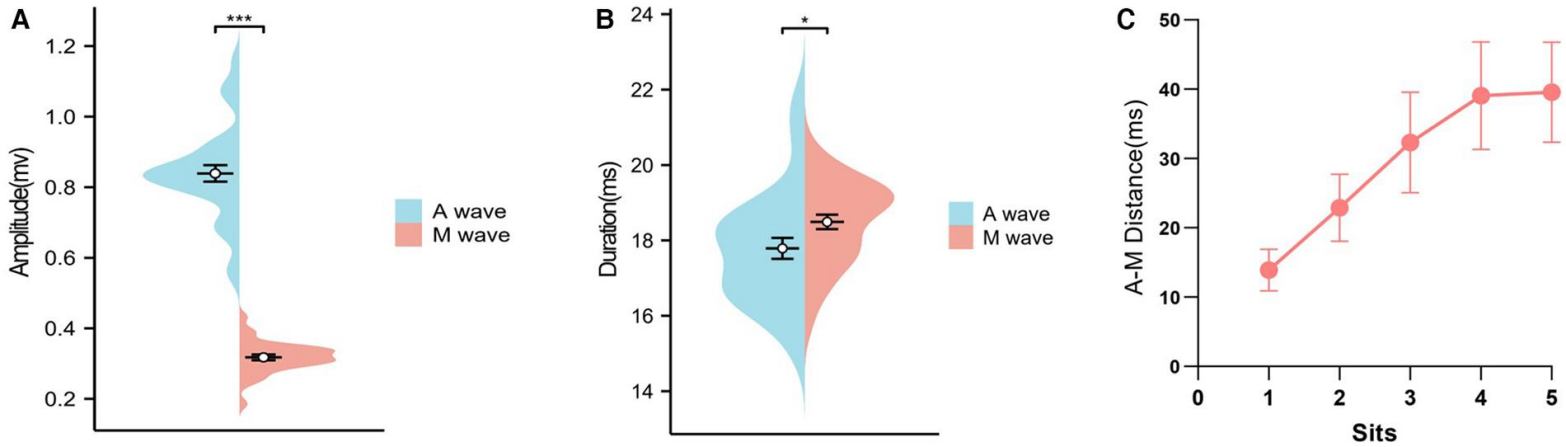
The characteristics of the double potential mapped in step 1. **A**: The amplitude difference between the A wave and the M wave. **B**: The duration difference between the A wave and the M wave. **C**: The increasing tendency of the A-M distance from the proximal to distal part of the curve mapped in step 1. A wave: potential from the left atrium. M wave: potential from Marshall.

Five points were selected for analysis from proximal to distal along the curve formulated in step 1 (Supplementary Figure S3). The amplitudes, durations, and distances of the two potentials of the bipotential recorded in step 1 were analyzed separately. The ahead potential of the double potential was deemed to be from the left atrium (A wave) and the latter one was deemed to be from Marshall (M wave). The amplitude of the M wave was lower compared to that of the A wave (0.84 ± 0.134 vs. 0.32 ± 0.046, *p* < 0.001), with a longer duration (18.41 ± 1.09, *P* = 0.019). The distance between the A wave and the M wave has an increasing tendency from the proximal to the distal site of the curve we mapped in step 1 (Figure 5).

### Comparison of procedure parameters between the two groups

The procedural parameters were compared between the two groups (Table 2). Both the total procedure time (*p* = 0.032) and total ablation time (*p* < 0.001) were significantly shorter in the QDM group than in the STSF group. Compared to the STSF group, the total mapping time was longer in the QDM group (*P* < 0.001), while the radiofrequency times were significantly shorter for ablating PVs, conducting roof line, and MI (*p* < 0.001). The total fluoroscopy time was not statistically different between the two groups.

**Table 2:** Comparison of procedure parameters between STSF and QDM.

Parameters	STSF group *N* = 36	QDM group *N* = 32	*P*-value
Total procedure time (min)	201 (165, 226)	176.5 (157.5, 223)	0.032
Total ablation time (min)	100 (87, 114)	85.5 (66, 112)	<0.001
Total mapping time (min)	14 (11, 17)	20.5 (15.5, 23)	<0.001
Total mapping time for potential from VOM (min)	-	8 (7, 11)	-
Total fluoroscopy time (min)	15 (11, 18.8)	14 (16, 19)	0.092
Total RF application time for ablating PVs (min)	45 (43, 55)	37 (32, 43)	<0.001
Total RF application time for ablating Roof Line (min)	7 (6, 9)	4 (4, 5)	<0.001
Total RF application time for MI (min)	23 (17, 25)	12.5 (9, 20)	<0.001

VOM, vein of Marshall; PVs, pulmonary veins; RF, radiofrequency; MI, mitral isthmus.

### The operative time and effects of each step in both groups

The median operation time of step 1 in the QDM group was 4 min. There were no differences in the operation time of EI-VOM between the two groups, and only 1 patient in the STSF group achieved conduction block of MI after that. Fewer patients in the QDM group required “V-shaped” and EASs ablation than those in the STSF group. Conduction block of MI was achieved in 71.9% of patients in the QDM group after a single line ablation, which was more than that in the STSF group (*P* = 0.017). Furthermore, with a shorter endocardial ablation time, the total number of ablation points was significantly lower in the QDM group compared to those in the STSF group (*P* < 0.001). Most importantly, after step 3, the QDM group exhibited a higher conduction block rate of MI than that in the STSF group (90.6% vs. 69.4%; *P* = 0.019). In step 4, the total number of ablation points with shorter ablation time (*P* < 0.001) was fewer in the QDM group than those in the STSF group (*P* < 0.001). Besides, the cumulative operation time was significantly shorter in the QDM group than that in the STSF group after step 4 (*P* = 0.021). Details of the comparison were shown in Table 3 and Supplementary Figure S4.

**Table 3:** Comparison of the time and effects of different steps in STSF and QDM.

Procedural outcome	STSF group *N* = 36	QDM group *N* = 32	*P*-value
Step 1: Outline the curve of VOM from the endocardial side			
Operation time (min)	-	4 (3, 5)	-
Step 2: EI-VOM			
Operation time (min)	14 (12, 15)	14 (12, 15)	0.764
Accumulated operation time (min)	13.5 (12, 15)	18 (16, 19)	<0.001
Conduction block of MI	1 (2.8)	0 (0)	-
Step 3: Endocardial ablation			
One line of “V sharp,” *n* (%)	13 (36.1)	23 (71.9)	0.017
Ablation after EASs mapping, *n* (%)	14 (38.9)	5 (5.6)	0.033
Total ablation points	19 (17.0, 21.8)	14 (12,16)	<0.001
Operation time (min)	16 (15, 19)	13 (11,16)	<0.001
Accumulated operation time (min)	30 (27.2, 33)	30 (27,33)	0.921
Conduction block of MI, *n* (%)	25 (69.4)	29 (90.6)	0.031
Step 4: Epicardial ablation			
Total ablation points	10 (6.3, 12.8)	4.5 (0, 6)	<0.001
Operation time (min)	8 (7, 10)	5 (4, 6)	<0.001
Accumulated operation time (min)	36 (30.5, 40.8)	31.5 (28.0, 36.8)	0.021
Conduction block of MI, *n* (%)	34 (94.4)	31 (96.8)	0.626

VOM, vein of Marshall; EI-VOM, ethanol infusion of the vein of Marshall; MI, mitral isthmus; operation time is expressed in minutes; and total number of ablation points is expressed as median (IQR); conduction block of MI, one line of V sharp, and ablation after EASs mapping were expressed by *n* (%); IQR: interquartile range; EASs: earliest activation sites.

### Complications

Only 4 patients experienced complications in this study. No patients suffered fatal complications. Two patients in the STSF group and one patient in the QDM group developed pericardial effusion but did not need pericardiocentesis. In the QDM group, one patient developed a hematoma due to femoral venous puncture.

## Discussion

The present study used the QDOT micro catheter to facilitate MI ablation in patients with PeAF. Furthermore, we modified the stepwise method for MI block proposed previously (7). Our main findings were as follows: (1) VOM potential could be mapped around MI from the endocardial surface using the QDOT micro catheter. These potentials characterized by bipotential with a higher sharp potential from LA and a lower sharp potential from VOM (Figure 2). (2) With fewer ablation points, the modified stepwise protocol increases the incidence of MI block after a single endocardial ablation line compared to the stepwise strategy. (3) The rate of conduction block of MI without epicardial ablation was higher in the QDM group than in the STSF group.

Additional linear ablation beyond PVI was recommended to reinforce the maintenance of sinus rhythm after AF ablation (3–5). However, the strategy has not been widely recognized and used due to the technical challenges. It was reported that incomplete lesions created anatomical substrates and could bring additional risks of atrial tachyarrhythmias (12). Mitral isthmus (MI) ablation is a key component and the most difficult step to achieve bidirectional block in a linear strategy due to its anatomical structure (6). The advent of new technologies and tools has increased the possibility of achieving MI bidirectional block, while variable block rates of MI are still reported in different studies (7–9).

The ligament of Marshall (LOM), which contains cardiomyocytes, adipose tissue, fibrous tissue, small blood vessels, and nerve tissue, lies on the epicardial surface of the MI area and increases its difficulty of ablation (13, 14). EI-VOM was reported to improve the success rate of MI block and AF ablation (3). We have also reported a novel stepwise catheter ablation method for MI that included EI-VOM, V-shape ablation from the endocardium, and ablation of KATs from the epicardium, obtaining a high bidirectional block rate of 98.3% (7). However, those methods involved extensive endocardial and epicardial ablation in the procedure (7–9). The imprecise ablation strategy increased the operational difficulty and risk.

Hwang et al. reported that the VOM potential could be recorded by an electrophysiological catheter into the VOM (10); however, the VOM potential was recorded from the epicardium. The QDM catheter is a novel catheter for very high power-short duration (vHPSD) ablation. It contains 3 micro-electrodes (0.086 mm^2^) located at the tip of the electrode at an angle of 60°. The clinical feasibility and safety of MI ablations using the QDM catheter have been demonstrated by previous clinical studies (11, 15). Dello Russo et al. demonstrated that microelectrode mapping using the QDM catheter revealed higher amplitude, longer duration, and more fractionated electrograms, thereby identifying potential pathology in suspected regions more effectively than standard bipolar electrode mapping (16). Therefore, the QDM catheter may be able to record the VOM potential from the endocardium and facilitate precise endocardial ablation.

In this study, we used the precise mapping capability of the embedded micro-electrodes in the QDM catheter to record the VOM potential from the endocardium, along which the endocardial ablation line was performed precisely. The bipotential recorded by the QDM catheter from the surface of the endocardium includes a higher sharp potential from LA and a lower sharp potential from VOM. We speculate that the sites on the endocardial surface where the VOM potential can be recorded likely correspond to the endocardial terminations of myocardial bundles connecting the endocardium and epicardium in the region surrounding the mitral isthmus. Therefore, the endocardial ablation line guided by the VOM potential recorded via the QDOT catheter is likely to maximize the likelihood of achieving conduction block of MI from the endocardial surface. Our research results thoroughly confirmed the above hypothesis. The results found that the modified stepwise method with the guidance of QDOT MICRO catheter increased the incidence of MI block after a single endocardial ablation line with fewer ablation lesions, thereby avoiding further epicardial ablation.

In this study, the QDM group had a shorter RF application time for ablating PVs and roof line ablation because the posterior wall of PVI and roof line ablation were performed using the vHPSD model (90W) of the QDM catheter in the QDM group, which improved ablation efficiency and achieved ablation effects in a shorter time. No significant difference was observed between the two groups in terms of complications, but vHPSD model may affect the occurrence of pericardial effusion and atrioesophageal fistula, which a larger sample size may be needed to confirm this.

### Limitation

Several important limitations of this trial warrant mention. First, this study is a single-center, randomized, controlled study with a relatively small sample size. Subsequently, the sample size needs to be expanded and a multi-center randomized controlled study should be conducted. Secondly, the present work has not specifically evaluated the long-term outcomes. Our research group followed all patients in our study, and the follow-up results will be published in future studies, providing insights into the short- and long-term prognosis.

## Conclusions

QDOT micro catheter can be used to map the potential of VOM from the endocardium, which facilitates guiding the endocardial MI linear ablation. The technique is able to reduce the endocardial ablation points and the chance of epicardial ablation with comparable complications.

## Data Availability

The raw data supporting the conclusions of this article will be made available by the authors, without undue reservation.
