# Supplementary material for: Modified stepwise method with the guidance of QDOT MICRO catheter for mitral isthmus ablation in patients with persistent atrial fibrillation
Source: Front Cardiovasc Med. 2026 Feb 26;13:1780048. doi: 10.3389/fcvm.2026.1780048 (PMC12979483; doi:10.3389/fcvm.2026.1780048)

**Supplement material**

**Supplement Figure 1**


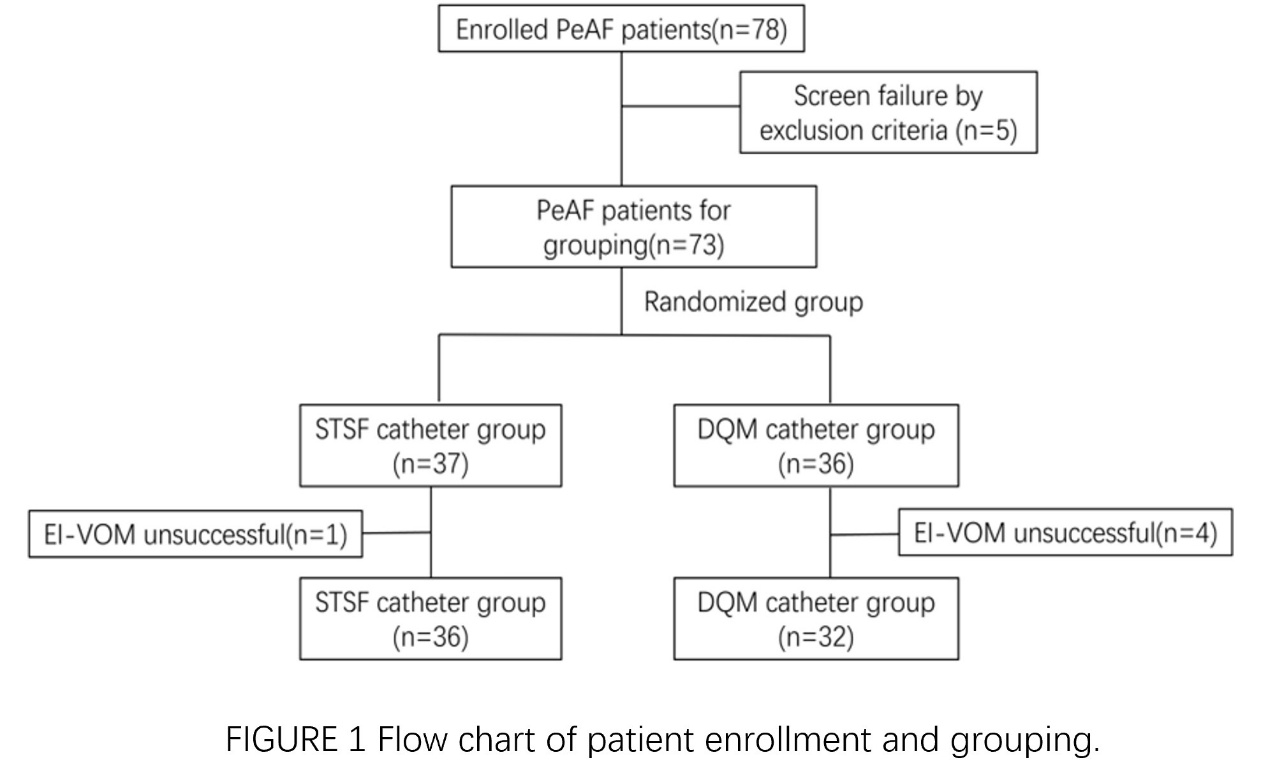


**Supplement Figure 2**


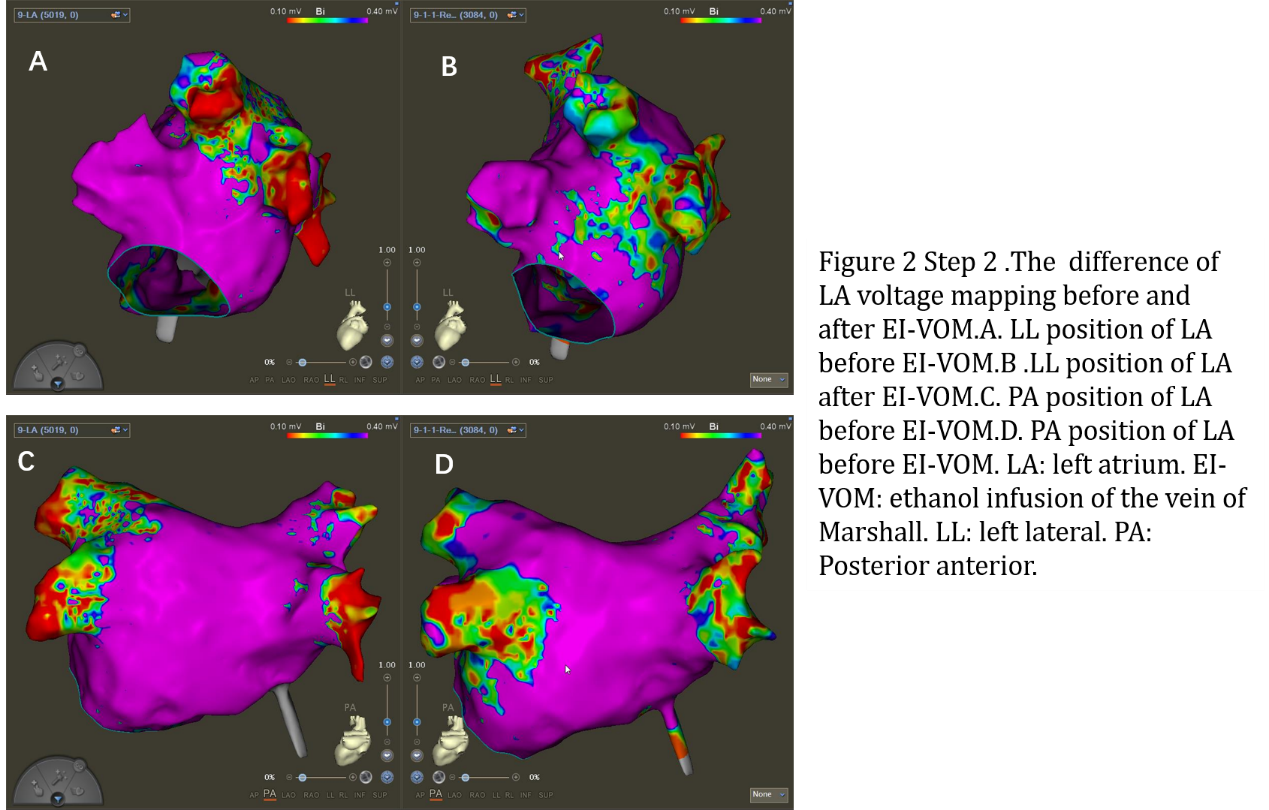


**Supplement Figure 3**


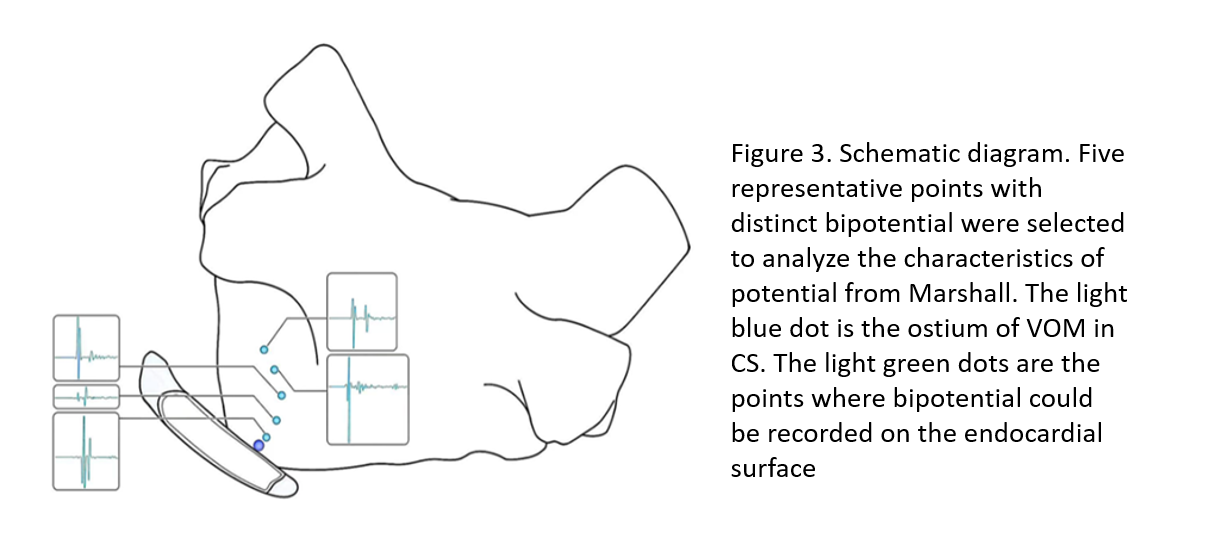


**Supplement Figure 4**


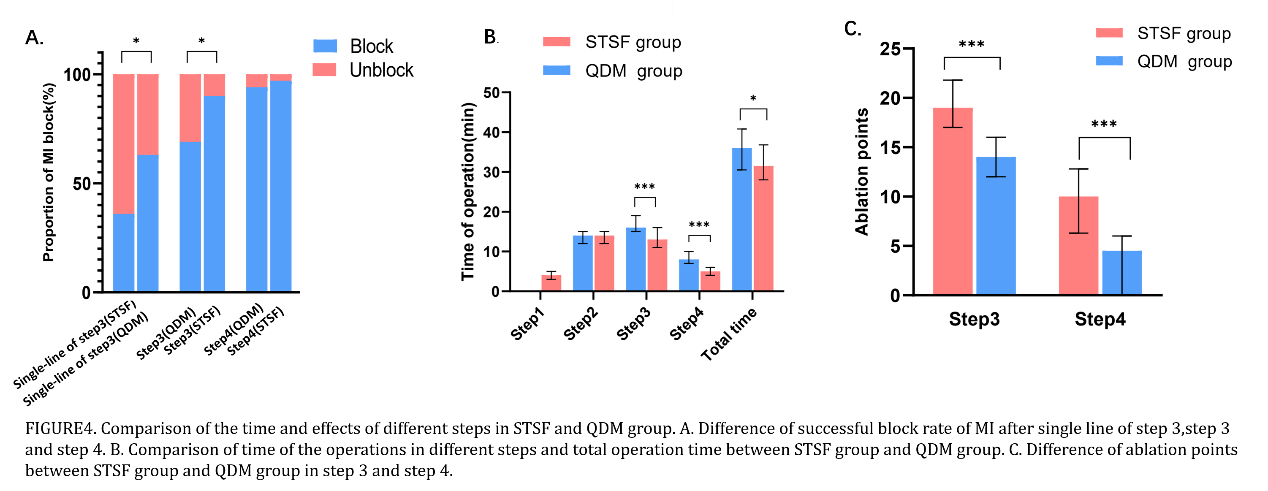

Supplement: Supplementary file 1 [file Datasheet1.docx]
